# A dynamic barrier: remodeling of the nuclear envelope during closed mitosis in malaria parasites

**DOI:** 10.1128/msphere.00999-24

**Published:** 2025-06-09

**Authors:** Sabrina Absalon

**Affiliations:** 1 Department of Pharmacology and Toxicology, Indiana University School of Medicine 12250, Indianapolis, Indiana, USA; Institut Pasteur de Montevideo, Montevideo, Uruguay

**Keywords:** *Plasmodium falciparum*, nuclear envelope dynamics, closed mitosis, ultrastructure expansion microscopy, nuclear pore complex, apicomplexan parasites

## Abstract

*Plasmodium falciparum*, the protozoan parasite responsible for the most severe form of human malaria, replicates through an unconventional mode of closed mitosis, where the nuclear envelope (NE) remains intact across multiple asynchronous nuclear divisions. This Full Circle minireview illustrates how a decade-long journey—from early electron microscopy observations of nuclear pore dynamics—has evolved into a broader investigation of NE composition, architecture, and regulation across the parasite life cycle. Advances in imaging, including ultrastructure expansion microscopy and cryo-electron tomography, revealed key features such as the bipartite microtubule organizing center, nuclear pore complex rosettes, and specialized NE scaffolds. Structure-guided and proteomic approaches identified divergent SUN-domain proteins, *Pf*SUN1 and *Pf*SUN2, as essential for NE integrity, genome stability, and chromatin positioning during schizogony. Hi-C analyses further uncovered species- and stage-specific chromatin organization, linking peripheral heterochromatin clustering to virulence gene regulation and life cycle progression. Despite lacking lamins, *Plasmodium*’s NE functions as a dynamic architectural hub that bridges chromatin, spindle microtubules, and organelle inheritance. Open questions remain about the full NE proteome, organelle-NE contact sites, and the possibility that mechanical deformation of the nucleus during red blood cell invasion could influence gene expression. These insights not only redefine *Plasmodium* cell biology but also position NE-associated components as attractive therapeutic targets. By coupling methodological innovation with conceptual inquiry, the study of NE dynamics in *Plasmodium* offers a powerful model for uncovering general principles of nuclear organization and adaptation in divergent eukaryotes.

## INTRODUCTION

When I first encountered the paper by Weiner et al. in 2011 (1), I was struck not just by the data, but by the questions it raised. Their use of focused ion beam scanning electron microscopy (FIB-SEM) to generate high-resolution 3D reconstructions of nuclear pore organization across the intraerythrocytic cycle of *Plasmodium falciparum* offered a tantalizing glimpse into the nuclear envelope (NE) of a parasite that replicates unlike any model eukaryote (2). The idea that this structure—a defining feature of eukaryotic cells—could remain intact throughout mitosis, while still accommodating spindle formation, organelle inheritance, and gene regulation, challenged my assumptions about cell division.

In my mSphere of Influence piece, I wrote about how that study catalyzed a deep interest in the mechanisms of NE remodeling during schizogony (3). Schizogony is a form of asexual reproduction in which the parasite undergoes multiple rounds of nuclear division without cytokinesis, followed by segmentation into daughter merozoites (4). At the time, relatively little was known about the molecular composition of the *Plasmodium* NE, and even less about how it adapted to the demands of the parasite’s rapid, asynchronous nuclear divisions. The field lacked tools, markers, and even conceptual frameworks to explore how the NE supported this unconventional form of mitosis. But what it did possess was a sense of mystery—and that was enough to set my lab on a path of discovery.

This minireview brings the story full circle. What began as curiosity about nuclear pores and chromatin has grown into a broader investigation of nuclear architecture and dynamics across *Plasmodium* life stages (5). Powered by methodological advances in live microscopy (6) and light microscopy like ultrastructure expansion microscopy (U-ExM)(7), proteomics approaches such as proximity labeling (8), and new genetic tools (9), we and others have begun to uncover the structural, functional, and regulatory elements that allow the NE to remain intact while supporting complex mitotic events. The NE is not just a passive boundary; it is a dynamic scaffold that spatially and temporally organizes division. Understanding how it works may reveal not only the unique biology of *Plasmodium* but also provide new therapeutic targets that can be exploited for our fight against malaria.

## CLOSED MITOSIS IN *PLASMODIUM*—A TRIGGER FOR DEEPER INQUIRY

**Fig 1:**
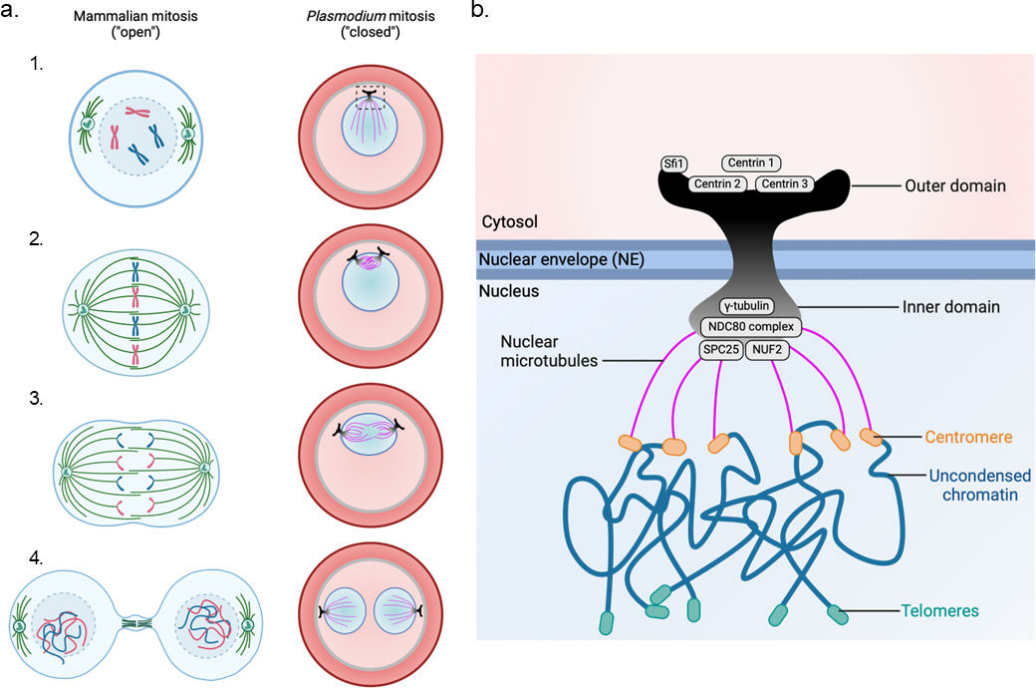
Divergent modes of mitosis and the centriolar plaque (CP) architecture in *Plasmodium*. (a) Comparison between mammalian open mitosis (left) and *Plasmodium falciparum* closed mitosis (right) during blood-stage replication (schizogony). *P. falciparum* is a haploid protozoan that undergoes asexual replication in human red blood cells through a unique process involving multiple rounds of nuclear division without immediate cytokinesis. (1) In open mitosis, the nuclear envelope breaks down, allowing cytoplasmic spindle microtubules to interact with kinetochores. In contrast, *Plasmodium* undergoes closed mitosis, with the nuclear envelope intact and spindle microtubules nucleated from the intranuclear side of the CP. (2) Chromosomes align at the metaphase plate in both systems; however, in *Plasmodium*, this occurs within an intact nucleus and involves 14 chromosomes that remain largely uncondensed throughout mitosis, never adopting the canonical X-shaped morphology observed in higher eukaryotes. (3) Anaphase and chromosome segregation proceed via nuclear microtubules. (4) Unlike higher eukaryotic cells that undergo a single round of synchronized mitosis followed by cytokinesis, *Plasmodium* blood-stage parasites undergo multiple rounds of asynchronous nuclear division within a shared cytoplasm. This results in a multinucleated schizont. Final daughter cell formation occurs through segmentation, a specialized cytokinesis process requiring the assembly of a dynamic multiprotein structure known as the basal complex. (b) Schematic of the *Plasmodium* CP, a bipartite microtubule-organizing center embedded in the nuclear envelope. The CP consists of an extranuclear outer domain (containing proteins such as Sfi1 and centrins) and an intranuclear inner domain (where γ-tubulin nucleates spindle microtubules). These microtubules connect to kinetochores via the NDC80 complex (including SPC25 and NUF2) to mediate chromosome segregation. Unlike canonical centrosomes, CPs lack centrioles and instead present a distinct architecture, comprising inner, core, and outer domains. Centromeres (orange) and telomeres (green) associate with the nuclear periphery, while chromatin remains uncondensed throughout mitosis.

*Plasmodium* parasites undergo closed mitosis, meaning the NE remains intact throughout multiple rounds of nuclear division (Fig. 1a). While this has been recognized for decades, what remains striking is the highly asynchronous nature of these divisions: nuclei that share the same cytoplasm behave autonomously, progressing through mitosis independently while residing only nanometers apart (10, 11). These divisions occur within a persistent NE, in contrast to the open mitosis of metazoans, where the NE breaks down as a prerequisite for spindle formation, chromosome attachment, and segregation (Fig. 1).

The features associated with closed mitosis and asynchronous division are evolutionarily tailored strategies that reflect the unique demands of *Plasmodium*’s intracellular replication (12). To understand how the NE accommodates such spatial and temporal complexity, we and others turned to advanced imaging approaches. Super-resolution microscopy and correlative electron tomography revealed that, rather than dismantling, the NE forms invaginations to accommodate spindle assembly within the nucleus (6).

A major barrier to studying NE dynamics in *Plasmodium* has been the absence of a reliable genetic NE marker. To address this, our lab developed the use of BODIPY TR ceramide as the first NE stain compatible with U-ExM (13). This innovation enabled high-resolution visualization of NE morphology throughout schizogony. When applied to parasites depleted of mini-chromosome maintenance (MCM) complex-binding protein—a member of the MCM complex required for DNA replication licensing—we observed dramatic phenotypes: anaphase chromatin bridges, malformed spindles, and single nuclei containing multiple microtubule structures, highlighting the consequences of compromised NE integrity during division.

Subsequent work using this tool has revealed even deeper links between nuclear organization and morphogenesis. For example, disruption of kinetochore components such as *Pf*NDC80 or *Pf*Nuf2 not only impairs spindle assembly and centromere integrity, but also leads to the physical uncoupling of the centriolar plaque (CP)—the parasite’s centrosome equivalent—from the NE, resulting in mispositioned apical complexes and the production of anucleate merozoites (14). These findings established a previously unrecognized role for the NE in coordinating nuclear anchoring and organelle inheritance.

Building on these insights, we and others revealed that the CP is a bipartite structure, composed of an extranuclear centrin-rich compartment and an intranuclear microtubule organizing center (MTOC), using a combination of advanced electron tomography and super-resolution microscopy (5, 6). Fig. 1 presents a schematic of the bipartite organization of the *Plasmodium* MTOC, highlighting its spatial relationship with the NE and its division of labor across the NE. These components are stably anchored to the NE and serve as key organizational hubs linking mitosis to parasite polarity and daughter cell formation. In this context, the NE is far more than a passive barrier—it acts as a dynamic scaffold that coordinates nuclear division, centrosome positioning, and intracellular architecture.

This evolving understanding prompted us to move beyond textbook cytology and ask: What molecular machinery enables the NE to remodel without rupturing? What signals regulate this remodeling? And how is NE integrity maintained under such rapid and repeated strain? The advent of U-ExM has been transformative in this pursuit, providing a method to visualize sub-organelle structures using light microscopy with nanoscale precision. Beyond simply resolving spindles and plaques, U-ExM has allowed us to begin constructing a holistic map of how the *Plasmodium* nucleus coordinates division with daughter cell formation. But these structural insights now demand functional interrogation: Which proteins scaffold these NE rearrangements? How is membrane tension resolved? And how are these systems integrated with the cell cycle? These questions continue to guide our investigations into this remarkable system.

Recent studies have uncovered several key components of the bipartite MTOC in *P. falciparum*, including three centrin isoforms (15) and the centrin-binding protein Sfi1 in the cytosolic compartment (16), and kinetochore-associated proteins such as NDC80, NUF2, and SPC25 in the nuclear compartment (17, 18). While canonical centrosomal scaffolds are missing (19), the presence of mitotic kinases and γ-tubulin suggests functional specialization adapted to closed mitosis (20, 21). The structural and molecular divergence of this MTOC highlights the need to define how such minimal and spatially split machineries coordinate mitotic progression in the parasite.

## FROM NUCLEAR PORE COMPLEX (NPC) CURIOSITY TO ROSETTE DISCOVERY

My initial fascination with NE dynamics in *Plasmodium* was closely tied to NPCs—not just their role in transport, but their enigmatic organization during schizogony. The Weiner et al. paper hinted at something tantalizing: NPCs were not static structures evenly sprinkled across the NE; they clustered, redistributed, and potentially linked to chromatin organization. It was an observation that raised more questions than answers.

At the time, we knew very little about the *Plasmodium* NPC. Only a handful of nucleoporins had been identified, and no comprehensive map of NPC composition or localization dynamics existed. The challenge of identifying NPCs in *Plasmodium* stemmed from their extreme divergence from canonical Opisthokont homologs, often lacking conserved motifs and domains. Proximal labeling combined with proteomic analyses, such as those described by Ambekar et al., enabled the identification of novel nucleoporins by exploiting their physical proximity to known NE proteins (8). These efforts were pivotal in assembling an initial parts list of the *Plasmodium* NPC.

Complementing these advances in composition, a 2024 cryo-electron tomography study of the NPC in *Toxoplasma gondii*, an apicomplexan parasite closely related to *Plasmodium*, provided the first *in-situ* structure of the complex at nanometer resolution (22). This study resolved the *in-situ* architecture of the *Toxoplasma* NPC at nanometer resolution and revealed that it lacks the canonical cytoplasmic nuclear basket observed in Opisthokont systems. Given the shared evolutionary lineage and the similarly compact NPC composition observed in *Plasmodium*, it is likely that the basket is also absent in this parasite. These findings emphasize how divergent and specialized apicomplexan NPCs have become, and as we continue to map their composition and behavior, it is clear that the *Plasmodium* NPC holds more surprises ahead.

To better understand NPC dynamics, we adapted the recombination-induced tag exchange (RITE) system to *Plasmodium* to track the turnover of NPC components (23). This innovative approach allowed us to distinguish old versus newly synthesized nucleoporins and directly observe their redistribution during mitosis. Yet, despite these breakthroughs, questions remain—particularly how new NPCs are incorporated into the NE during division, a process that remains elusive in this parasite.

A turning point came with the development of U-ExM, which provided an unprecedented view of nanoscale assemblies using light microscopy. For the first time, we could visualize the NPC’s organization *in situ* with sub-organelle clarity. This tool opened the door to a new level of inquiry, and our lab dove in.

In *P. berghei*, we discovered that NPCs do not just scatter or cluster randomly—they form organized rosettes around the CP (the parasite’s centrosome equivalent) as nuclei prepare to divide (23). These rosettes, consisting of 4–10 NPCs in a symmetric array, circumscribe the site of intranuclear spindle formation. This kind of structured assembly around MTOCs had not been reported in other eukaryotes, suggesting a specialized adaptation in *Plasmodium* to coordinate nucleocytoplasmic transport with mitotic events.

**Fig 2:**
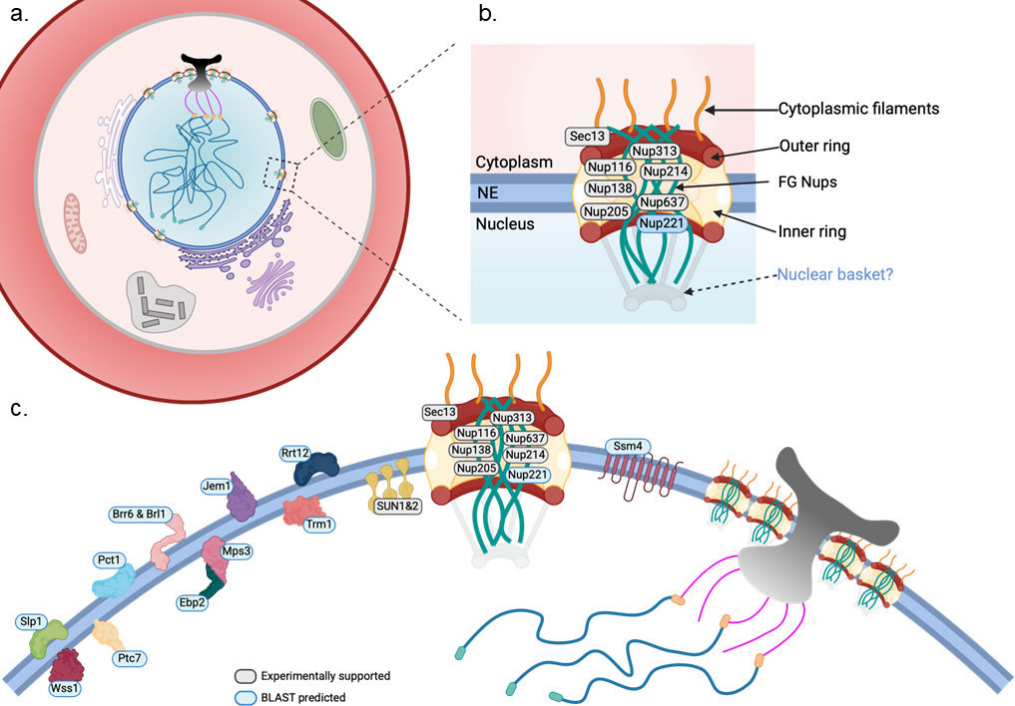
Nuclear envelope (NE) organization and nuclear pore complex (NPC) architecture in asexual Plasmodium blood-stage parasites. (a) Schematic overview of a *P. falciparum* parasite upon entry into mitosis and cell division (schizogony) within the human red blood cell. The parasite contains a single nucleus surrounded by a NE, which houses the bipartite centriolar plaque (CP) and hundreds of NPCs. NPC biogenesis occurs upon mitosis, and the newly formed pores are distributed among daughter nuclei following each nuclear division. NPCs are not evenly spaced but form a distinctive rosette-like cluster around the CP, a spatial organization that is maintained throughout mitosis. In the cytoplasm, the endoplasmic reticulum (ER), Golgi apparatus, and food vacuole (site of hemoglobin digestion). Young parasites (ring; early trophozoites) contain a single mitochondrion and a non-photosynthetic plastid organelle called the apicoplast, both of which possess their own genome. (b) Model of the *P. falciparum* NPC, showing individual nucleoporins (Nups) organized into cytoplasmic filaments, outer ring, FG-repeat-containing channel proteins, and inner ring components. Nups shown in light gray-colored boxes are experimentally supported, while those in blue boxes are predicted by BLAST homology. The presence of a nuclear basket remains uncertain; in the related apicomplexan *T. gondii*, cryo-electron tomography (cryoET) has revealed that a canonical nuclear basket is absent, raising the possibility that *Plasmodium* may share this architectural feature. (c) Zoomed-in view of the NE in *P. falciparum*, illustrating multiple NPCs embedded in the NE and their spatial proximity to the CP. Both experimentally supported and BLAST-predicted NE-associated proteins are depicted, with consistent color-coding. SUN-domain proteins (SUN1 and SUN2) are shown to bridge the NE, potentially linking chromatin to the nuclear periphery. Transmembrane proteins such as Ssm4 and Slp1 may contribute to NE architecture or chromatin anchoring. Notably, *Plasmodium* lacks a nuclear lamina, a scaffold structure found in many eukaryotes, which may influence how chromatin is tethered to the NE and how nuclear integrity is maintained during closed mitosis. NPCs remain clustered near the CP, underscoring a potential functional connection between nuclear transport, mitotic microtubule organization, and chromosome segregation.

While the dynamic regulation of NPC number during schizogony was initially observed in the Weiner et al. study, our application of U-ExM allowed us to quantify these changes with greater precision. We found that NPC number peaks before each nuclear division and is halved during karyokinesis, indicating that NPCs are not built *de novo* for each daughter nucleus but inherited and redistributed (Fig. 2). The RITE system revealed turnover of specific components such as Nup221—older and newly synthesized copies coexisted within the same NPCs, showing that even in “closed” mitosis, the NPC is not static. Fig. 2 provides a schematic view of the *Plasmodium* NPC, highlighting its modular organization and the experimentally identified components such as Nup221, as well as homologs predicted by rBLAST searches. If permitted, we include cryo-ET data from *Toxoplasma* to visualize the apparent divergence from the classical NPC scaffold.

These discoveries challenged our assumptions. Rather than passive channels, NPCs in *Plasmodium* appear to be modular and responsive, reorganizing in space and composition as needed. Their arrangement near the CP may contribute to regulating mitotic entry—perhaps by concentrating factors that initiate MTOC duplication or coordinate nuclear division, especially given the asynchronous nature of mitosis during schizogony.

While overlapping transport routes have now been demonstrated in mammalian NPCs using live-cell super-resolution imaging (24), it remains unclear whether this organizational principle applies to the structurally divergent NPCs of apicomplexan parasites, which may exhibit distinct modes of remodeling and regulation.

The structural plasticity of these complexes, combined with their apparent integration into mitotic architecture, underscored a broader lesson: in *Plasmodium*, the NE does not merely retain its integrity during mitosis—it appears flexible and responsive, potentially adjusting its components in coordination with division. While we cannot conclude that the NE is actively remodeling, the involvement of NE-associated proteins in maintaining structural integrity warrants further investigation, particularly in connection with MTOC dynamics.

## TETHERED BY THE ENVELOPE—SUN PROTEINS AND SPATIAL COORDINATION

One of the enduring mysteries of *Plasmodium* cell biology is how the parasite preserves both NE integrity and chromatin architecture throughout rapid, repeated rounds of nuclear division—without canonical lamin proteins (25). While NE persistence during closed mitosis is expected, what remains less understood is how *Plasmodium* ensures nuclear positioning and genome organization in the absence of a classical nuclear lamina or characterized inner/outer membrane scaffolds. Studies from Dr. Karine Le Roch’s group and others have shown that peripheral heterochromatin clustering near the NE is tightly correlated with gene expression control, but the proteins that anchor chromatin or connect the NE to the cytoskeleton remain largely unidentified (26–28). This challenge is illustrated in Fig. 2, where the parasite nucleus must coordinate multiple architectural demands—including microtubule nucleation, NPC remodeling, and envelope continuity—all within the physical constraints of a closed mitosis.

Recent comparative genomics and structure-based prediction tools like AlphaFold and DA LI have helped uncover previously hidden components of this architecture (29, 30). A landmark study led by Sayers identified a divergent SUN-domain protein (*Pb*SUN1) in *P. berghei*, along with its interacting partner *PbALLC1* (31). These proteins localized to the NE in stage V gametocytes. Based on structural and spatial analysis, the authors proposed that SUN1 may be required to capture MTOCs onto the nuclear membrane, a function critical for organizing spindle microtubules during sexual differentiation. However, this study did not investigate blood-stage parasites.

Further work from Tewari’s group in *P. berghei* male gametogenesis showed that a related SUN1 protein forms a complex with *Pb*ALLAN (32). Using U-ExM, the SUN1–ALLAN complex was shown to bridge the intranuclear spindle poles with cytoplasmic basal bodies at the NE. Disruption of this complex via gene knockout produced “ghost” microgametes that formed axonemes but lacked nuclear DNA—highlighting the essential role of NE–MTOC connectivity in genome segregation.

Building on these sexual-stage studies, a new publication has now demonstrated the essential roles of SUN-domain proteins in blood-stage parasites. A study identified and characterized two SUN-domain proteins in *P. falciparum: Pf*SUN1 (PF3D7_1215100) and *Pf*SUN2 (PF3D7_1439300) (33). Both proteins contain conserved SUN domains and coiled-coil motifs and localize to the nuclear periphery during schizogony. Importantly, depletion of either SUN1 or SUN2 disrupted nuclear segregation and cytokinesis, producing abnormal schizonts with defective chromatin partitioning and membranous whorls at the NE.

Each protein appears to play a distinct role. *Pf*SUN1 was found to be critical for the DNA damage response (DDR), as its depletion blocked phosphorylation of the *Pf*H2A histone variant following X-ray irradiation, a key step in initiating repair. *Pf*SUN2 did not affect DDR directly but was shown to associate with heterochromatin at the NE, implicating it in chromatin organization. These functions echo those of SUN proteins in other apicomplexans like *T. gondii*, but also reveal unique, parasite-specific adaptations to support mitotic fidelity in the absence of lamins.

These findings close a major gap in our understanding of NE function in asexual stages. SUN proteins are now emerging as essential mediators of nuclear architecture and integrity across *Plasmodium* life cycle stages. Figure 2 provides an overview of NE-associated proteins, both identified and predicted, including SUN-domain proteins, Sfi1, and other potential scaffolds.

In summary, SUN-domain proteins in *Plasmodium* serve as critical organizers of nuclear dynamics—from anchoring MTOCs and maintaining genome integrity to coordinating the NE with spindle architecture and chromatin. Their divergence from canonical LINC complexes, essential roles in blood- and sexual-stage development, and involvement in DDR make them promising candidates for therapeutic targeting—particularly given their absence in humans and their essential roles in parasite proliferation.

## SHIFTING PARADIGMS WITH METHODS—HOW TOOLS RESHAPED THE QUESTIONS

Throughout this journey, one theme has remained constant: our understanding of *Plasmodium* NE dynamics has been driven as much by the tools we have developed as by the questions we have asked. In many ways, the field’s conceptual leaps have paralleled its technological ones.

It began with FIB-SEM, which provided high-resolution snapshots that first hinted at non-random NPC distribution and possible links to chromatin. But while powerful, FIB-SEM could not capture the dynamic, life-stage-specific rearrangements that underlie the parasite’s biology. What followed was the advent of U-ExM, a method that did not just allow us to see more clearly, but to think differently. By physically expanding the parasite while preserving its ultrastructure, U-ExM allowed volumetric reconstructions of entire schizonts and high-resolution visualization of subnuclear compartments. It was through U-ExM that we could uncover NPC rosettes, track spindle assembly, and define the bipartite MTOC *in situ*. Complementary tools continued to push the envelope. Electron tomography and correlative light and electron microscopy resolved intranuclear spindle compartments and verified CP anchoring to the NE. Cryo-electron tomography revealed the structural divergence of the apicomplexan NPC and the absence of canonical scaffolds like the cytoplasmic basket. Each technique offered a new vantage point on the same biological question: how does the NE scaffold and coordinate division in a parasite that breaks many of the eukaryotic rules?

To these imaging breakthroughs, we now add a genomic complement: Hi-C. Originally developed to map 3D genome architecture, Hi-C has transformed our understanding of chromatin organization at the nuclear periphery. In *P. falciparum* and *P. knowlesi*, Hi-C revealed that subtelomeric virulence genes—such as the var family—are tethered to heterochromatic clusters at the nuclear periphery. These repressive “neighborhoods” support monoallelic expression, a key mechanism of antigenic variation and immune evasion. Chromosomes with internal virulence genes form long-range loops that deliver them to these clusters, emphasizing how genome topology is functionally encoded (26).

Hi-C also revealed conserved centromere and telomere clustering, forming rosette-like configurations likely maintained by NE contacts (34). In the absence of lamins, SUN-domain proteins such as *Pf*SUN1 and *Pf*SUN2 are hypothesized to act as anchoring tethers, organizing chromatin at the periphery. Disruption of these SUN proteins, as recent work has shown, leads to loss of chromatin organization, nuclear deformability, and defective DDR.

Importantly, Hi-C data have highlighted species- and stage-specific differences in genome architecture. Pathogenic species like *P. falciparum* (a major human malaria parasite) and *P. knowlesi* (a zoonotic species capable of infecting humans and macaques) exhibit tighter peripheral virulence gene clustering than less virulent relatives, while comparisons across developmental stages—such as blood-stage schizonts (undergoing asexual replication in red blood cells) and mosquito-stage sporozoites (the transmissible form injected by the mosquito)—reveal dynamic reorganization of chromatin–NE interactions during the parasite life cycle.

Taken together, U-ExM, cryo-ET, and Hi-C have converged to redefine what is visible—and therefore, what is askable. Questions that once seemed out of reach—How is chromatin organized in 3D? How do NPCs remodel? What anchors the MTOC to the NE?—are now within experimental grasp. Perhaps most exciting is that these methods are now being adopted across the field, catalyzing discovery at every level of *Plasmodium* biology—from the nanometer scale of nuclear pores to the megabase loops of virulence genes.

## FULL CIRCLE AND FUTURE DIRECTIONS—UNRAVELING THE NUCLEAR NEXUS

When I wrote my mSphere of Influence piece in 2020, I was standing at the edge of a field filled with unknowns. The work of Weiner et al. had revealed tantalizing hints of nuclear pore reorganization during *Plasmodium* development, but the underlying mechanisms—and even many of the relevant players—remained unknown. My questions then were foundational: How does the NE stay intact through multiple rounds of division? What machinery allows it to accommodate mitotic spindle assembly? Where are the regulators that coordinate nuclear and cytoplasmic events in the absence of canonical checkpoints?

Since then, what began as a curiosity about NPC dynamics has evolved into a broader investigation of nuclear architecture and cellular coordination. Together with others in the field, we have shown that *Plasmodium* has retooled conserved mechanisms—like the LINC complex—into highly divergent systems. We have uncovered specialized structures, from intranuclear spindle compartments and NPC rosettes to bipartite MTOCs that anchor to the NE. We have come to understand the NE not as a passive boundary, but as a dynamic, stage-specific platform that scaffolds chromatin, anchors microtubules, and links division to organelle inheritance.

And yet, some of the most exciting questions lie ahead.

Chief among them is the molecular composition and regulation of NE-associated complexes. While SUN-domain proteins, nucleoporins, and kinetochore components have now been mapped (35), we still lack a full proteomic inventory of the NE across the *Plasmodium* life cycle. Even less is known about how these components interact with cell cycle regulators, or how their remodeling is coordinated with the demands of replication and transmission.

Another frontier lies in understanding the functional interfaces between the NE and other organelles. The NE is contiguous with the endoplasmic reticulum, but its consistent proximity to key organelles—particularly the apicoplast and mitochondrion—suggests additional, unexplored coordination. A recent FIB-SEM study (36) revealed that the apicoplast remains closely associated with the CP (MTOC) throughout schizogony. During nuclear division, the apicoplast extends toward the CP, implying that the MTOC may serve as a positional landmark for organelle segregation. These observations raise the possibility of physical membrane contact sites or signaling hubs that synchronize mitotic progression with apicoplast inheritance—an area ripe for investigation.

Finally, the functional consequences of nuclear architecture remain a rich and underexplored terrain. How does the NE shape gene expression programs across life stages? To what extent does chromatin positioning at the nuclear periphery drive parasite virulence or developmental transitions? Can we target these scaffolding mechanisms therapeutically, exploiting their divergence from host counterparts?

A question of particular personal interest is whether mechanical deformation of the nucleus during invasion could modulate gene expression in *Plasmodium*. During red blood cell invasion, the parasite undergoes extreme morphological changes, including a dramatic elongation and reshaping of the nucleus to navigate the narrow invasion cleft (37). Despite the absence of lamins, the NE remains intact—likely stabilized by SUN-domain proteins and cytoskeletal elements—raising the intriguing possibility that nuclear architecture may serve not only as a mechanical buffer but also as a regulatory platform. While hardwired, stage-specific transcriptional programs generally govern *Plasmodium* gene expression, the potential for non-canonical mechanosensitive responses—or structural cues influencing chromatin organization and organelle positioning—remains a provocative avenue for future investigation.

As new imaging, proteomic, and genomic tools continue to expand what we can visualize and manipulate, we find ourselves in a uniquely powerful position—not just to describe *Plasmodium* nuclear biology, but to explain it mechanistically. In doing so, we may uncover organizing principles that extend beyond malaria, illuminating how eukaryotic cells build flexible, resilient systems to thrive under evolutionary pressure.
